# Zygomycosis in a renal allograft recipient

**DOI:** 10.4103/0971-4065.50679

**Published:** 2009-01

**Authors:** G. Lakshminarayana, R. Rajesh, G. Kurian, V. N. Unni

**Affiliations:** Department of Nephrology, Amrita Institute of Medical Sciences and Research Center, Cochin, India

**Keywords:** Immunosuppression, renal transplantation, zygomycosis

## Abstract

Invasive fungal infections can cause considerable morbidity and mortality in immunocompromised patients. Zygomycosis is a type of invasive fungal infection with a rapid course and grave prognosis. Renal transplant recipients with concomitant diabetes mellitus are most susceptible to this infection. We report here a case of disseminated zygomycosis (*Rhizopus* sp.) in a renal allograft recipient with posttransplant diabetes mellitus (PTDM). This is the first reported case of zygomycosis caused by *Rhizopus* species.

## Introduction

Infections continue to be a significant cause of morbidity and mortality in renal transplant recipients. Mycotic diseases in these patients are often fatal because they are difficult to diagnose and treat. Newer, potent immunosuppressive agents and the presence of underlying medical conditions such as diabetes mellitus (DM) increase the risk for fungal infections.

## Case Report

A 48 year-old female was detected to have end stage renal disease and was initiated on hemodialysis. After three months on hemodialysis, she underwent a spousal donor renal transplantation (immunosupression was accomplished with Cyclosporine A, azathioprine, prednisolone, and induction with Dacluzimab). She became oliguric and graft function worsened on the second postoperative day. Graft biopsy done on the third postoperative day showed interstitial hemorrhage and endothelitis with fibrin thrombi suggestive of accelerated acute rejection. She was treated with IV Methyl prednisolone (500 mg × 3 doses) and the azathioprine was replaced with Mycophenolate Mofetil (MMF). Her graft function did not improve and she became anuric; hemodialysis was started. Repeat graft biopsy showed only partial improvement and she remained anuric and dependent on dialysis. She was given seven doses of antithymocyteglobulin (THYMOGAM Horse, 500 mg) with CMV prophylaxis (Valganciclovir); Cyclosporine A was withdrawn and Sirolimus was started.

There was gradual improvement in the urine output over the next three weeks and hemodialysis was discontinued. She was continued on MMF, Sirolimus, and prednisolone; her serum creatinine level improved to 1.2 mgdL on subsequent follow-up over four weeks. She developed diabetes mellitus (PTDM) two weeks after transplantation.

**Figure 1:**
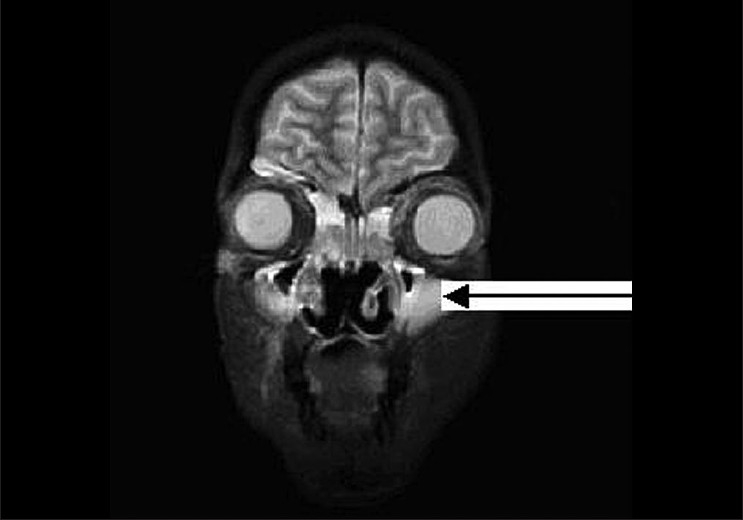
MRI scan showing swollen mucosa in the bilateral maxillary sinus with fluid collection (arrow)

**Figure 2:**
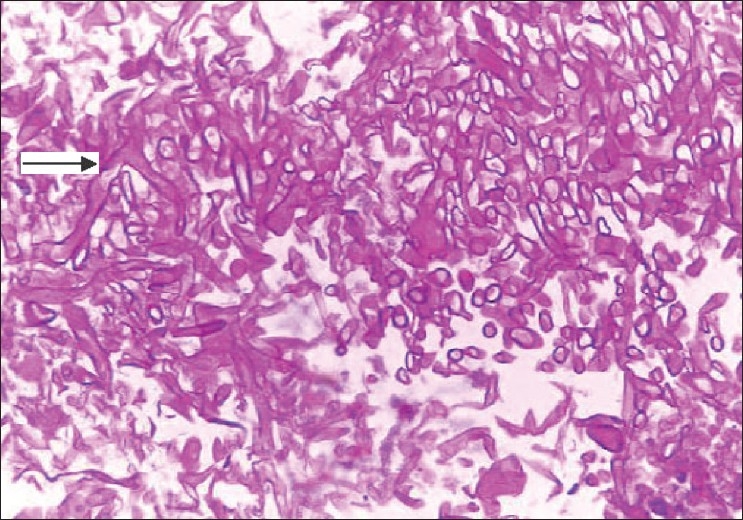
Histology of sinus mucosa showing extensive necrosis with branching aseptate hyphae and a few distorted hyphae (arrow)

**Figure 3:**
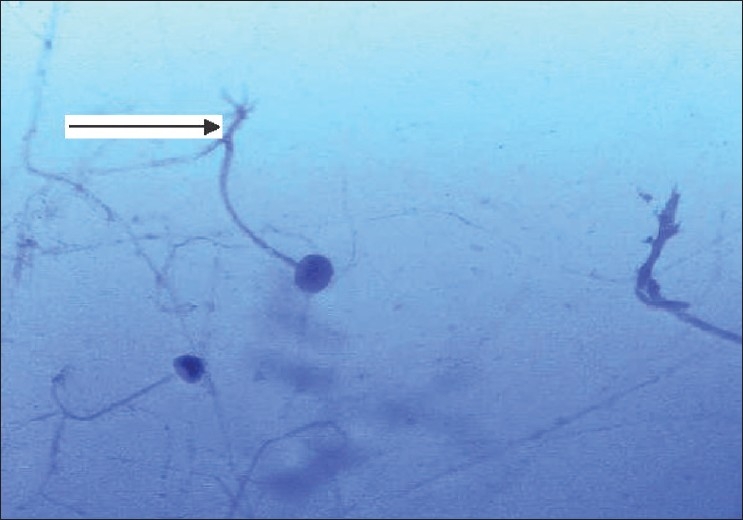
Smear of sinus mucosal culture showing branching aseptate hyphae (arrow) suggestive of *Rhizopus species* (Lactophenol blue Calcofluor stain)

She was readmitted six weeks after transplantation with epistaxis and high-grade fever of two days' duration with tenderness over the maxillary sinuses. MRI scans revealed swollen mucosa of the maxillary and frontal sinuses with fluid collection in both maxillary sinuses [Figure 1] and a 3 cm-sized lesion in the frontal lobe of the brain. A chest X-ray showed nonhomogenous opacities in the right middle and lower zones. Histological examination of the sinus mucosa revealed extensive angioinvasion and necrosis with branching aseptate hyphae and a few distorted hyphae [Figure 2] suggestive of zygomycetes, which on culture was identified as *Rhizopus* [Figure 3]. As the lungs, paranasal sinuses, and the brain were involved, a diagnosis of disseminated zygomycosis was made. Immunosuppression was reduced and she was treated with Amphotericin B (1 mg/kg/day) and extensive debridement of the nasal and sinus mucosa. Her condition worsened progressively; ventilatory and inotropic support was started. She succumbed to her illness in about two weeks although she had good allograft function until the end.

## Discussion

Opportunistic fungal infections occur in 6–10% of all recipients of solid organ transplants with a very high mortality (70–100%) being reported from the Indian subcontinent.[1] Reports of systemic mycoses in the transplant population from Western countries reveal an incidence of 1.4–9.4%. The prevalence of systemic mycoses was reported to be 6.6% in a study from Southern India, similar to that in North India.[1] Despite ongoing improvements in immunosuppressive therapy and surgical techniques, fungal infections remain a significant cause of morbidity and mortality in organ transplant recipients throughout the world with very few reports of successful treatment.[1–10]

### Pathogenic fungi

The list of fungal pathogens causing disease in renal allograft recipients has grown to over 250 species.[4] However, over 90% of the medically important fungal infections belong to the fungi imperfectii such as aspergillosis, candidiasis, mucormycosis, coccidiodomycoses, histoplasmosis, and paracoccidiodomycoses.[1,4] Whereas candidiasis is the most common infection and can be effectively treated, there has been a recent rise in angioinvasive fungal infections such as zygomycosis, which are associated with a high mortality.[1–6]

### Zygomycosis

Zygomycosis is a collective term referring to infections caused by fungi of the order *mucorales*, which includes pathogens such as *Rhizopus, Mucor, Rhizomucor,* and *Absidia*.[1] Although most of the cases reported in renal transplant recipients are due to the *Mucor* species; the species remain unidentified in some reports. These ubiquitous fungi are found in decaying vegetative and organic matter. They have minimal, intrinsic pathogenicity but can initiate grave and often-fatal infections in certain clinical conditions with compromised host defenses. Studies have shown that the Mucor subtype is more angioinvasive than others and has the worst prognosis. Risk factors that predispose individuals to fungal infections include diabetes mellitus, chronic liver disease, operative technical errors, re-exploration, retransplantation, the duration of transplant procedure, postoperative haemodialysis, and the use of monoclonal antibodies and other potent immunosuppressives such as ATG.[1,4,6] Although studies show increased incidence of infections (bacterial and viral) following antirejection therapy with methyl prednisolone, OKT-3, and ATG, no clear increase in mucormycosis has been reported.[5]

Zygomycosis presents with various clinical manifestations,[5] depending on the portal of entry and the predisposing risk factors of the patient. The five major clinical forms are: (1) rhinocerebral, (2) pulmonary, (3) abdomino-pelvic and gastric (gastrointestinal), (4) primary cutaneous, and (5) disseminated.

Rhinocerebral is the most frequently encountered form of the disease observed primarily in patients with diabetic acidosis. Patients typically present with a history of fever, unilateral facial pain or headaches, nasal congestion, epistaxis, visual disturbances, and lethargy. Physical examination may reveal periorbital cellulitis, proptosis, and loss of extraocular muscle movement. These lesions are frequently accompanied by cranial nerve palsy involving the III, IV, and VI nerves.

Pulmonary zygomycosis presents with a history of fever, cough, hemoptysis, chest pain, and increasing shortness of breath. Primary pulmonary zygomycosis tends to occur in patients with hematological malignancy or profound neutropenia and in those who have been on steroid therapy. Pulmonary infections have been the most common mode of presentation of zygomycotic infections among diabetic renal allograft recipients, although extrapulmonary sites such as rhinocerebral, central nervous system, renal allograft and musculoskeletal, cutaneous, and gastrointestinal infections have all been reported.[1–10]

Gastrointestinal zygomycosis typically presents with a history of abdominal pain or distention, dyspepsia, nausea and vomiting, diarrhea, and hematochezia. This form usually results when a patient who is malnourished or has renal failure, ingests the organism. Infection results in necrotic ulcerations with ischemia and gangrene of the stomach and colon.

Cutaneous zygomycosis may be primary, resulting from direct inoculation of the organism into the disrupted integument. Primary disease is due to local trauma or inoculation whereas secondary disease is due to hematogenous dissemination to the skin. Secondary cutaneous infection is observed with widely disseminated zygomycosis because of hematogenous seeding.

The disseminated form generally has its source in the lungs and spreads hematogenously to the central nervous system. Patients typically present with a history of headache, fever, visual disturbances, and changes in mental status. Physical examination may reveal lethargy, obtundation, coma, sudden onset of focal neurologic deficits, and necrotic ulcerations on the respiratory tract mucosa or the skin. Disseminated zygomycosis in patients with hematological malignancies begins in the lungs and spreads to the CNS, producing infarction and abscess. It can also spread to the liver, spleen, kidney, heart, and skin.

### Laboratory diagnosis

#### Direct microscopy:

(a) Scrapings, sputum, and exudates should be examined using 10% KOH and Parker ink or Calcofluor mounts, (b) Tissue sections should be stained with Hematoxylin and eosin (H and E). Broad, infrequently septate, thin-walled hyphae that often show focal bulbous dilations and irregular branching are characteristics of Zygomycosis.

#### Culture:

Primary isolation media such as Sabouraud's dextrose agar are used. As most zygomycetes are sensitive to cycloheximide, this agent should not be used in culture media. Zygomycetes are usually fast-growing fungi with white-to-grey or brownish, downy colonies characterized by primitive coenocytic (mostly aseptate) hyphae seen on culture. Asexual spores include chlamydoconidia, conidia, and sporangiospores contained in sporangia borne on simple or branched sporangiophores. Sexual reproduction is isogamous producing a thick-walled, sexual, resting spore called a zygospore.

#### Histology:

Diagnosis is made by histological examination of the infected tissue and demonstration of characteristic, broad, aseptate hyphae branching irregularly at right angles. These features can distinguish them from the slender hyphae of Aspergillus, which have regular dichotomous branching and frequent septae.[5] The characteristic feature of *Mucor* infection is vascular invasion with thrombosis involving large and small arteries, which results in infarction and necrosis of the infected organ.[5] Renal histological examination in patients who have renal involvement typically shows evidence of vasculitis with infarction and necrosis of the kidneys as well as parenchymal infiltration with neutrophils.[1,2,5]

### Treatment

Early tissue diagnosis and prolonged treatment with antifungal agents such as amphotericin B are required for the eradication of these tissue-invasive infections.[1–5] Newer generation agents such as lipid-based amphotericin B preparations have allowed higher doses of the drug to be delivered within short periods, leading to enhanced efficacy and reduced toxicity. The dose recommended for treatment of mucormycosis is amphotericin B (0.5–1 mg/kg/day) or lipid-based (lipid complex, colloidal dispersion, and liposomal) amphotericin B (1–5 mg/kg/day); the recommended cumulative dose is 2–2.5 g over 6–8 weeks.[1,2,5]

## Conclusion

Zygomycosis is a type of invasive fungal infection that has a rapid downhill course and grave prognosis, and hence, needs early diagnosis and aggressive management. This is the first reported case of zygomycosis caused by *Rhizopus* species in renal allograft recipients. Disseminated type is rare, but this form has a very high mortality inspite of aggressive chemotherapy and surgical management. A high degree of suspicion would lead to an early diagnosis and prompt initiation of treatment. The risk of fatal opportunistic infections such as zygomycosis is the price to pay when attempts are being made to save the graft with aggressive immunosupression.
